# The interactive effects of drought and heat stress on photosynthetic efficiency and biochemical defense mechanisms of *Amaranthus* species

**DOI:** 10.1002/pei3.10092

**Published:** 2022-10-13

**Authors:** Mmbulaheni Happiness Netshimbupfe, Jacques Berner, Chrisna Gouws

**Affiliations:** ^1^ Unit for Environmental Science and Management North‐West University (Potchefstroom Campus) Potchefstroom South Africa; ^2^ Centre of Excellence for Pharmaceutical Sciences (Pharmacen™) North‐West University Potchefstroom South Africa

**Keywords:** *Amaranthus* sp., chlorophyll *a* fluorescence, photosystem II

## Abstract

Drought and heat stress are major abiotic stress factors that limit photosynthesis and other related metabolic processes that hamper plant growth and productivity. Identifying plants that can tolerate abiotic stress conditions is essential for sustainable agriculture. *Amaranthus* plants can tolerate adverse weather conditions, especially drought and heat, and their leaves and grain are highly nutritious. Because of these traits, amaranth has been identified as a possible crop to be grown in marginal crop production systems. Therefore, this study investigated the photochemical and biochemical responses of *Amaranthus caudatus*, *Amaranthus hypochondriacus*, *Amaranthus cruentus*, and *Amaranthus spinosus* to drought stress, heat shock treatments, and a combination of both. After the six‐leaf stage in a greenhouse, plants were subjected to drought stress, heat shock treatments, and a combination of both. Chlorophyll *a* fluorescence was used to evaluate the photochemical responses of photosystem II to heat shock while subjected to drought stress. It was found that heat shock and a combination of drought and heat shock damages photosystem II, but the level of damage varies considerably between the species. We concluded that *A. cruentus* and *A. spinosus* are more heat and drought‐tolerant than *Amaranthus caudatus* and *Amaranthus hypochondriacus*.

## Graphical abstract

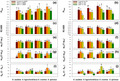

## 1 INTRODUCTION

Abiotic stressors such as drought and heat stress remain the main limiting factors for the development and productivity of many crop species. It is also expected to be the same for *Amaranthus*, an important herbaceous medicinal plant and inexpensive nutritional vegetable (Al‐Mamun et al., 2016; Sarker & Oba, 2018). This highlights its importance for crop diversification in dry areas of southern Africa for future agricultural systems (Chivenge et al., 2015; Modi, 2006; Oyodele, 2000).

Many parts of South Africa and the SADC region are experiencing major water shortages and temperatures above 40°C during summer (Baudoin et al., 2017; Jansen van Rensburg et al., 2007; Vogel & van Zyl, 2016). The more frequent occurrence of these extreme weather conditions challenges crop production in South Africa. *Amaranthus* is widely regarded as a drought‐tolerant crop due to its ability to recover after a long period of severe drought stress (Sarker & Oba, 2018). *Amaranthus* also has a high tolerance level to various environmental stressors such as soil salinity, drought, heat, diseases, and pests (Omami & Hammes, 2006; Sairam & Tyagi, 2004). It can also tolerate soil pH ranging from 4.5 to 8 due to mycorrhizal associations that enable *Amaranthus* to maximize the use of rare nutrients (Chaudhari et al., 2009; Paland & Chang, 2003). Therefore, *Amaranthus* is regarded as a prospective crop for marginal land and semi‐arid regions (Bhattacharjee & Mukherjee, 2006; Bianco et al., 2000; Steckel et al., 2004).

In many cases, heat stress occurs as a result of short‐term exposure to sublethal temperatures. Such exposure may result in irreversible damage to photosynthetic apparatus, cellular and subcellular structures, and their functions. The altered tertiary and quaternary structures of membrane proteins enhance membrane permeability and electrolyte leakage (EL). Consequently, the enhanced EL can lead to a decrease in membrane thermostability which is accompanied by plant tissue senescence (Farooq et al., 2008; Kigel et al., 1977; Savchenko et al., 2002; Yamane et al., 1998; Yan et al., 2011, 2013). However, some plants have developed heat avoidance and tolerance mechanisms to survive heat stress. Plants tolerate heat stress by synthesizing heat shock proteins and enhancing the production of secondary compounds through protective biochemical mechanisms (Fares et al., 2010; Sharkey et al., 2001; Vickers et al., 2009; Wang et al., 2004). Such mechanisms involve producing volatile organic compounds, which play a crucial role in alleviating cellular membrane damage and oxidative stress induced by heat stress (Loreto et al., 2004; Rennenberg et al., 2006).

Plants may also develop specific morphological characteristics, such as altered leaf shapes (Paland & Chang, 2003). On the contrary, altered physiological processes assist plants in tolerating heat stress better. Osmoprotectants like proline help plants tolerate drought and heat stress by stabilizing the structures of DNA, cellular membranes, and protein complexes. Several studies have indicated that proline is responsible for scavenging reactive oxygen species (ROS) and other free radicals (Bohnert et al., 1995; Giberti et al., 2014; Rejeb et al., 2014; Smirnoff & Cumbes, 1989). It is also responsible for regulating intracellular redox potential, energy transfer, and energy storage (Giberti et al., 2014; Kaur & Asthir, 2015).

Combined drought and heat stress have a deleterious effect on photosynthesis of both C3 and C4 sensitive plants and are more severe than those individual effects (Abdulaziz & Alhaithloul, 2019; Mingnan et al., 2017; Sehgal et al., 2017; Zhou et al., 2019; Zhu et al., 2021). Photosynthesis is a major component of plant growth that is extremely sensitive to drought and heat stress (Ashraf & Harris, 2013; Chaves et al., 2009; Hopkins & Hüner, 2006; Wise et al., 2004). However, the severity and duration of drought and heat stress determine how various photosynthetic parameters respond (Ashraf & Harris, 2013; Cicevan et al., 2016; Mathobo et al., 2017). Generally, drought and heat stress affects photosynthesis through stomatal closure and reduction of the cellular water potential. Consequently, the chloroplast is deprived of atmospheric CO_2,_ which compromises the structural integrity of the photosynthetic machinery. CO_2_ availability is, therefore, a rate‐limiting factor of photosynthesis. The rate of photosynthesis is determined by the concentration of intracellular CO_2_, which supplies CO_2_ at the carboxylation site for assimilation in the chloroplast. However, plants use resources efficiently by maintaining intracellular CO_2_ levels at a transition zone without excess electron transport and carboxylation capacity (Camejo et al., 2005; Wahid et al., 2007).

Chlorophyll *a* fluorescence was used to study the interactive effects of drought and heat stress and a combination of both on the PSII photochemical efficiency of *Amaranthus*. Because the O‐J‐I‐P transient is sensitive to environmental stress (Krüger et al., 1997; Stirbet & Govindjee, 2011; Tsimilli‐Michael et al., 1998, 1999), the JIP‐test is used to interpret the original fluorescence measurements (Strasser & Tsimilli‐Michael, 2001; Strasser et al., 2000). Analysis of the O‐J‐I‐P fluorescence rise using the JIP‐test (Strasser & Strasser, 1995; Srivastava, Strasser, & Govindjee, 1999; Strasser et al., 1999, 2000) provides a platform to measure the flux of energy passing through the photosystems and assessing the photosynthetic performance of the plants and PSII function (Strasser et al., 2004; Tsimilli‐Michael & Strasser, 2008, 2013).

The effect of drought and heat stress on photosynthetic parameters has been extensively studied and different plants were considered in this respect (Bahrami et al., 2019; Goltsev et al., 2016; Kalaji et al., 2017; Lazár, 2015; Lichtenthaler et al., 2005; Maliba et al., 2018; Osipova et al., 2019; Oukarroum et al., 2012; Singh et al., 2019; Xue et al., 2017). However, the comparative analysis of the interactive effect at different intensities of drought and heat stress on photosynthetic parameters' response and other biochemical parameters across temperature regimes remained untouched in the cited studies. Therefore, this article explores the interactive effect of drought and heat stress and a combination of both on photochemical efficiency. Furthermore, this study seeks to demonstrate that *A. cruentus* and *A. spinosus* have better adaptive mechanisms to tolerate drought and heat stress, and are more suitable for semi‐arid regions.

## 2 MATERIALS AND METHODS

### 2.1 Plant material and growth conditions

The experiment was performed in a greenhouse with a day/night air temperature of 26/18°C and a 13 h day photoperiod and a dark period of 11 h. Fluorescent growth tubes (130 μmol [photon] m^−2^ s^−1^) were used to supplement light in a greenhouse. A RHT03 humidity and temperature sensor with a single wire digital interface were used to monitor the temperature inside the greenhouse.

*Amaranthus sp*. (*A. caudatus*, *A. hypochondricus*, *A. cruentus and A. spinosus*) seeds were hand sown into 10 cm diameter pots containing hygromix substrate. A total of 12g of 6‐month slow‐release fertilizer containing 17 nitrogen: 11 phosphorus: 10 potassium: 2 manganese oxide: TE (Osmocote® Pro) was added at various medium levels to each pot. The pots were arranged in a randomized complete block design with three replications for each species and treatment. Seedlings were thinned when they reached the 2nd leaf stage and only one plant was reserved. Plants were manually watered on alternate days.

At the six‐leaf stage after germination, drought stress (10% field capacity) was induced by withholding water for 7 days. Plants were subjected to different temperature regimes ranging from 30 to 40°C. Plants at 26°C were used as the control group. The main experiments were repeated three to five times (*n*), with three replicates for each species.

### 2.2 Chlorophyll *a* fluorescence measurements and analysis of OJIP curves

The kinetics of the polyphasic prompt fluorescence rise, indicating *Q*_A_ reduction, were measured *in vivo* using the M‐PEA fluorimeter (Hansatech Instrument Ltd, King's Lynn) on 1 h dark‐adapted leaves. Measurements were taken at four different spots on the adaxial surface of 3 fully developed canopy leaves of three plants per treatment. Chlorophyll *a* fluorescence measurements were recorded after illumination by a red actinic light of 3000 μmol (photon) m^−2^ s^−1^ provided by three light‐emitting diodes with a 5 mm diameter focus spot and 12‐bit resolution in 1 s. Measured photo‐induced transient readings were analyzed by M‐PEA Plus data analyzer version 1.10 software (Hansatech Instrument Ltd, M‐PEA Plus, King's Lynn). The M‐PEA fluorimeter data points were set at 0.02 ms to 0.05 ms for the initial fluorescence O step, intermediates steps J at 2 ms, I at 30 ms, and peak P step at 300 ms. The curves were plotted on a logarithmic time‐scale and exhibited a series of steps between the initial step O (*F*_o_, when all the RCs of the PSII were open), and the maximum step P (*F*_m_, when all RCs of the PSII were fully closed or reduced). The maximal fluorescence (*F*_m_) and the minimal fluorescence (*F*_o_) were used to calculate the *F*_v_/*F*_m_ ratio, which is related to the quantum yield of the PSII photochemistry (Govindjee, 2004). The JIP‐test was used to calculate the prompt fluorescence transient induced by the first pulse of the red light according to the equations of the JIP‐test. Parameters such as the density of active *Q*_A_ reducing the PSII RCs per leaf cross‐section (RC/CS_o_), and the specific fluxes per active RC of the PSII, could then be derived (Strasser et al., 2004, 2010; Tsimilli‐Michael & Strasser, 2013; Yusuf et al., 2010) (Appendix A).

The kinetic analyses of the prompt fluorescence transient kinetics were evaluated by calculating the difference in the relative variable fluorescence to present ∆*V* (expressed as *V* = f[t]) curves between the OK and OJ steps, by normalizing as follows: *V*_OK_ = (*F*_t_ − *F*_0_)/(*F*_K_ − *F*_0_), as fluorescence data were normalized between O (0.02 ms) and K (0.3 ms) revealing the Δ*V*_L_‐band, *V*_OJ_ = (*F*_t_ − *F*_0_)/(*F*_J_ − *F*_0_), as fluorescence data were normalized between O (0.02 ms). The difference between Δ*V*_OK_ and Δ*V*_OJ_ transients, relative to the control treatment were assessed and plotted as the difference in kinetics Δ*V*_OK_ = *V*_treatment_ − *V*_control_ and Δ*V*_OJ_ = *V*_treatment_ − *V*_control_ (Yusuf et al., 2010). The ∆*V* bands (∆*V*_L_, ∆*V*_K_) were revealed on the curves at specific time points corresponding to the limitation in the photosynthetic electron transport chain.

### 2.3 Measurement of electrolytic conductivity

Membrane leakage was measured using the method of Sullivan (1971), Wisniewski et al. (1997), and Al Busaidi and Farag (2015). Briefly, leaf disks (7.5 mm diameter) from the youngest fully expanded leaves on each treated plant (4 weeks old) were punched out with a cork borer on a paper towel at different time intervals (before treatment and after 30, 60, and 120 min of heat treatment). The leaf disks were immediately submerged in separate test tubes containing 10 ml of sterilized ultrapure water. Hereafter, rinsed three times to remove electrolytes that initially leaked from the damaged cells on the edge of the leaf disks. The discarded water was replaced with 10 ml of fresh sterilized ultrapure water. The tubes were loosely covered with aluminum foil and the samples were placed in the dark for 24 h. The electrical conductivity (EC) meter (Primo 5, HANNA Instruments) was calibrated before use with the conductivity standard solution to 1.41 mS/cm. The sensor of the EC meter was rinsed with sterilized ultrapure water (between samples), after which the initial EL readings were measured. Tubes were closed and autoclaved at 121°C at a pressure of 103 kPa for 20 min to dissociate all cellular cytosols into a solution. After cooling to room temperature, the final EC measurements were taken and recorded as total ionic leakage. Determination of the EL was calculated as an injury index percentage at 100°C, using the following formula:

$$\left[1-\left(\frac{\text{Final}‐\text{Initial}}{\text{Initial}}\right)\times 100\right]$$

at the ratio of the initial EC to the final EC per time point.

### 2.4 Determination of proline content

Samples were extracted using a cold extraction procedure by homogenizing 0.02 g fresh leaf weight in 400 μl ethanol: water (40:60 v/v). The resulting mixture was stored at −20°C for future use. The samples were centrifuged at 14000 *g* for 5 min and 500 μl supernatant was pooled with a pipette and used for proline analyses (Carillo et al., 2008). To the 500 μl of sample extract, 1000 μl reaction mixture (ninhydrin 1% [w/v] in acetic acid 60% [v/v], ethanol 20% [v/v]) was added. The sealed tubes were incubated at 95°C in a block heater for 20 min after which they were centrifuged at 11,200 *g* for 1 min. The tube contents were transferred to a 1.5 ml cuvette and its absorbance was recorded at 520 nm against the blank in the spectrophotometer (CE1011 1000 Series, Cecil Instruments). A standard curve was constructed using the proline standard to determine the proline concentration in each sample. The proline content in the leaf extracts was calculated for each treatment using the following equation:

$$\text{Proline}=\left(\frac{\;{\mathrm{Abs}}_{\text{extract}}-{\mathrm{Abs}}_{\text{blank}}}{\text{Slope}}\right)\times \frac{{\mathrm{Vol}}_{\text{extract}}}{{\mathrm{Vol}}_{\text{aliquot}}}\times \frac{1}{\mathrm{FW}}$$

### 2.5 Determination of relative water content

A 1.5 x 1.5 cm square block was used to measure when cutting leaf disks to determine the leaf relative water content. The fresh leaf weight (FW) was measured immediately, after which the leaf disks were subsequently rehydrated by submerging them in distilled water for 24 h in Petri dishes until they reach full turgidity. The samples were reweighed (TW) after the leaf disks were removed from the water and blotted with a dry paper towel to remove surface water. The leaf disks were dried at 70°C to the point of brittleness for 48 h and the corresponding dry weights (DW) were determined. The relative water content (RWC) was calculated for each treatment using the following equation:

$$\mathrm{RWC}\;\left(\%\right)=\left(\frac{\mathrm{FW}\left(g\right)-\mathrm{DW}\left(g\right)}{\mathrm{TW}\left(g\right)-\mathrm{DW}\left(g\right)}\right)\times 100$$

### 2.6 Statistical analysis

The data were analyzed using SigmaPlot version 12.0 (Systat Software, Inc.) software. The normality test (Shapiro–Wilk) one‐way analysis of variance (ANOVA) was applied to all data in order to test the effects of drought and heat stress at each temperature regime level. Comparison among means was determined through the Fisher LSD method at *p* ≤ 0.05. The data in the figures and tables were expressed as mean ± standard deviation.

## 3 RESULTS

### 3.1 Transient fluorescence curves

**FIGURE 1:**
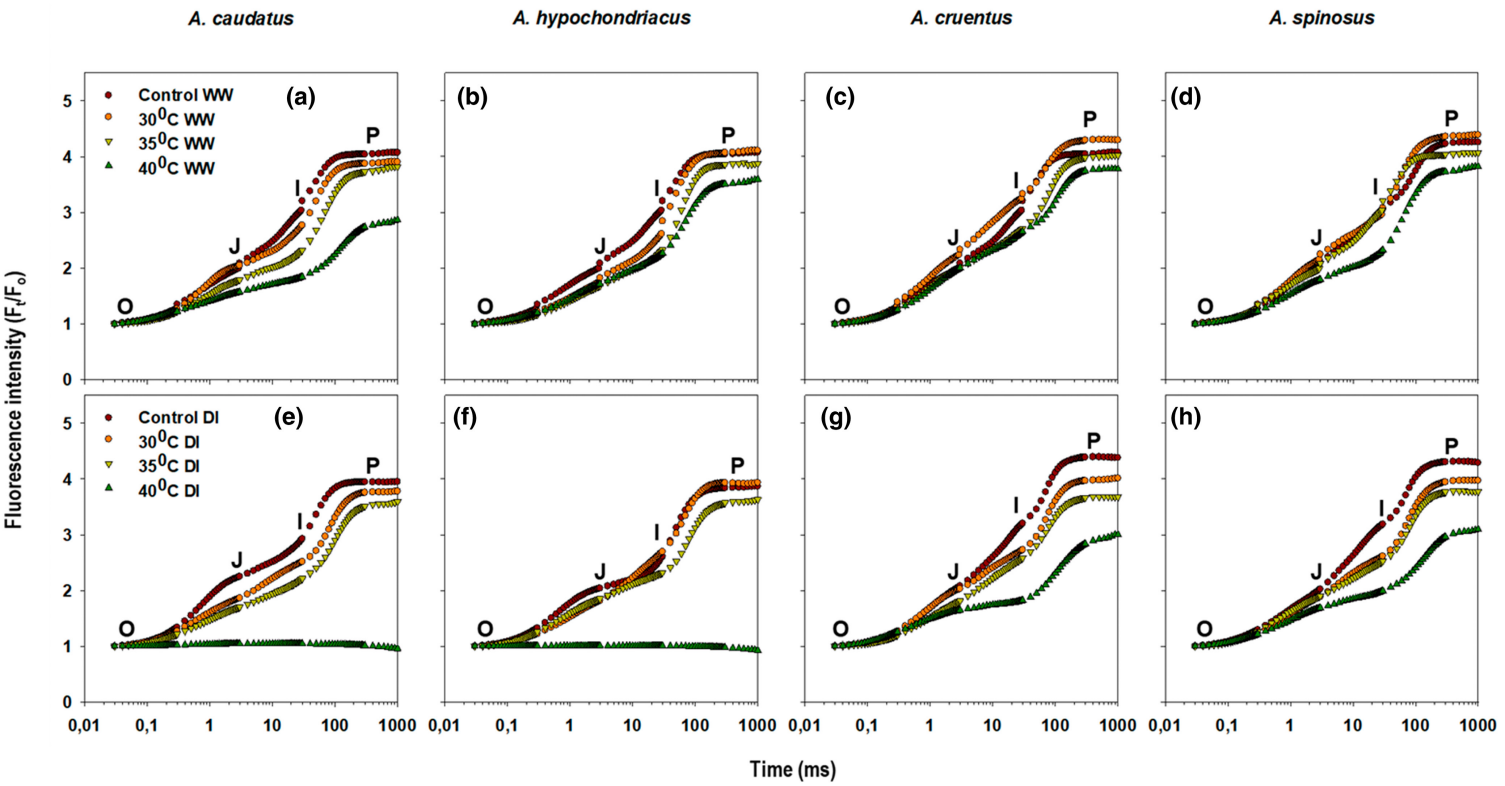
The average chlorophyll *a* fluorescence transient (OJIP) exhibited by dark‐adapted leaves of *Amaranthus* plants treated with 30, 35, and 40°C temperature regimes under well‐watered (WW) (a–d) and water‐stressed (DI) conditions (e–h), normalized at 0.03 ms and plotted on a logarithmic time scale.

The transient fluorescence curves (OJIP) were analyzed to show the overall impact of drought and heat stress on four *Amaranthus* species. The averages of the raw fluorescence transient data of the dark‐adapted amaranth leaves were plotted on a logarithmic time scale from 0.01 ms to 1000 ms and the values are expressed as *F*_t_/*F*_0_ (Figure 1). There were no significant (*p* ≤ 0.05) changes in the fluorescence intensity of the OJIP transient at the single turn‐over phase (0.01 –2 ms) under drought and heat stress. All tested *Amaranthus* species showed a slower rise in combined stress and reached a much lower fluorescence peak (Figure 1a–h). Both *A. caudatus* and *A. hypochondriacus sp*. showed a more pronounced decline in the fluorescence intensity of the OJIP transient at 35 and 40°C temperature regimes and under drought stress, relative to *A. cruentus* and *A. spinosus* (Figure 1a–h). However, *A. caudatus* and *A. hypochondriacus* showed a significant (*p* ≤ 0.05) increase in fluorescence intensity of the OJIP transient when subjected to a 30°C temperature regime and drought stress (Figure 1a,b,e,f).

Conversely, the fluorescence intensity for *A. cruentus* and *A. spinosus* was significantly (*p* ≤ 0.05) higher at 35 and 40°C temperature regimes (Figure 1c,d and g,h). However, these plants also had a lower fluorescence intensity at 30°C. The combined effect of drought and heat stress on the fluorescence intensity for *Amaranthus* species was much more pronounced at 35 and 40°C temperature regimes compared to 30°C for both species.

### 3.2 The difference in relative variable fluorescence

#### 3.2.1 ∆ *V*_L_‐Band

**FIGURE 2:**
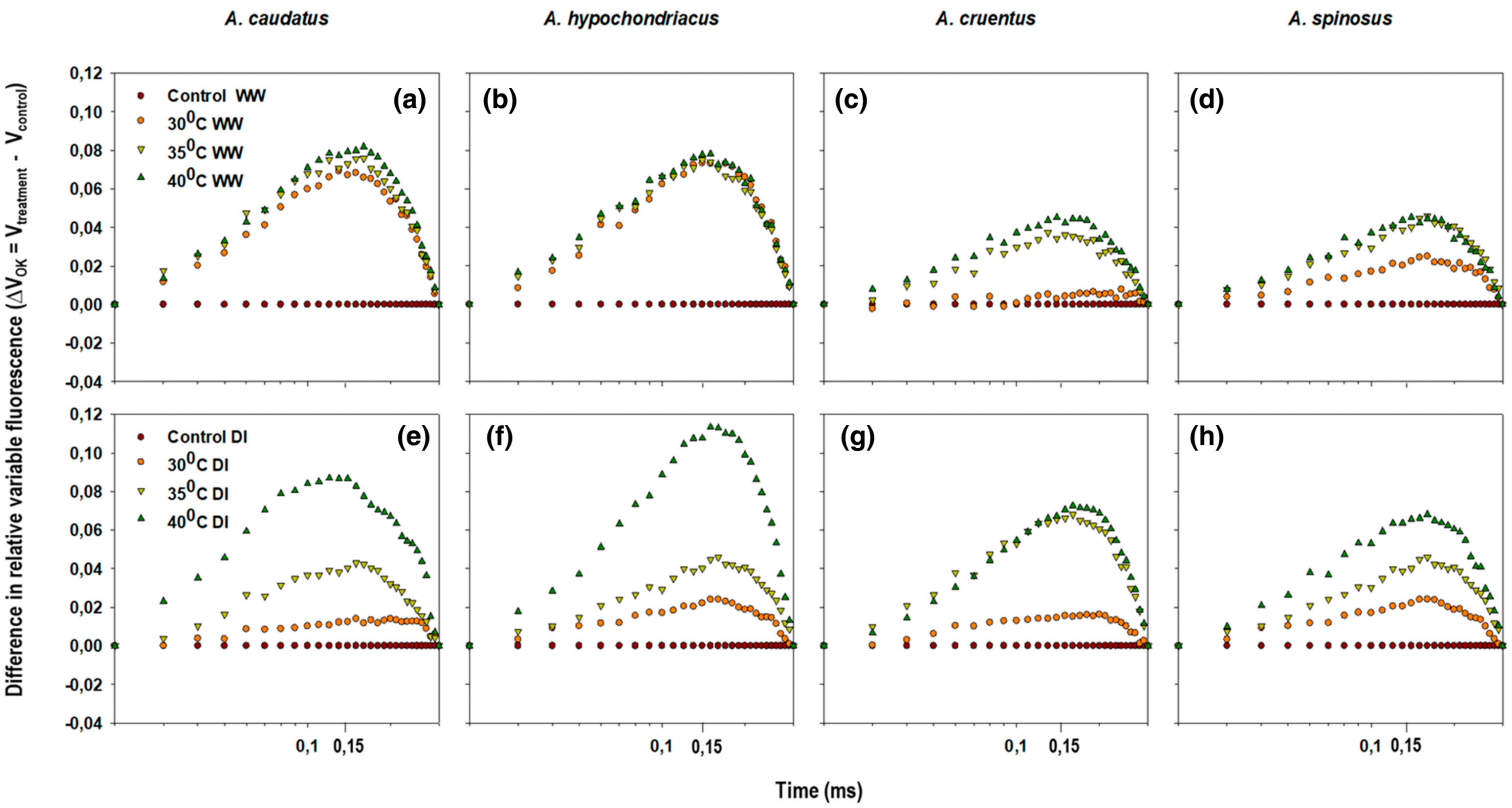
Changes in the difference of the relative variable chlorophyll *a* fluorescence transients (∆*V*_t_) treated with 30, 35, and 40°C temperature regimes under well‐watered (WW) (a–d) and water‐stressed (DI) conditions (e–h). ∆*V*_OK_ = *V*_treatment_–*V*_control_.

To reveal hidden differences between treatments, fluorescence data were double normalized between *F*_o_ (0.03 ms) and *F*_k_ (0.3 ms) steps, as *V*_OK_ = (*F*_t_−*F*_o_)/(*F*_k_−*F*_o_), and plotted as variable kinetics ∆*V*_OK_ = *V*_treatment_–*V*_control_ to reveal the ∆*V*_L_‐band. All *Amaranthus* species treated with drought, heat, and a combination of both stressors showed a positive ∆*V*_L_‐band (0.15 ms). Consequently, the appearance of a positive ∆*V*_L_‐band indicated decreased energetic groupings of the PSII units (Oukarroum et al., 2007; Strasser et al., 2004, 2007; Yusuf et al., 2010; Zhu et al., 2005). Among other species, the highest amplitude of the variable fluorescence was observed in *A. caudatus* and *A. hypochondriacus* treated with 30, 35, and 40°C temperature regimes under well‐watered and drought‐stress conditions (Figure 2a,b,e,f). Similarly, when these plants were treated with a 40°C temperature regime and combined stressors, the size of the variable fluorescence amplitude was increased significantly (*p* ≤ 0.05). The amplitude of the variable fluorescence was significantly lower (*p* ≤ 0.05) in *A. cruentus* and *A. spinosus* (Figure 2c,d,g,h).

### 3.3 ∆ *V*_K_‐Band

**FIGURE 3:**
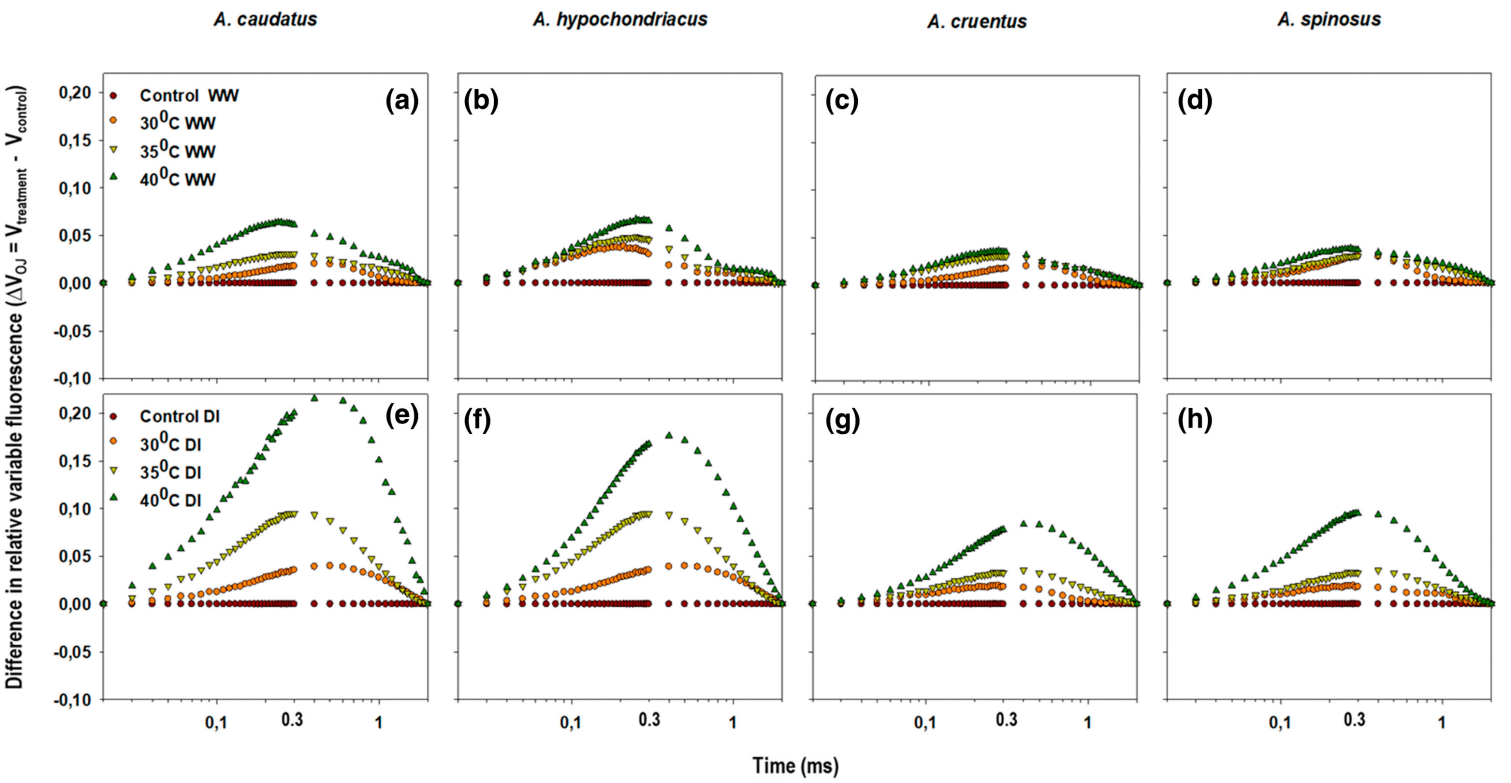
Changes in the difference of the relative variable chlorophyll *a* fluorescence transients (∆*V*_t_) treated with 30, 35, and 40°C temperature regimes under well‐watered (WW) (a–d) and water‐stressed (DI) conditions (e–h). ∆*V*_OJ_ = *V*_treatment_–*V*_control_.

The ∆*V*_K_‐band was revealed when the fluorescence data were normalized between the *F*_o_ (0.03 ms) and *F*_J_ (2 ms) steps, as *V*_OJ_ = (*F*_t_–*F*_o_)/(*F*_J_–*F*_o_), and plotted as variable kinetics ∆*V*_OJ_ = *V*_treatment_–*V*_control_. The positive ∆*V*_K_‐band (0.3 ms) was visualized in *Amaranthus* species treated with drought and heat stressors. The exhibition of a positive ∆*V*_K_‐band for all treatments was an indication that the oxygen‐evolution complex (OEC) was inactivated or the functional PSII antenna size was increased (Oukarroum et al., 2012; Strasser et al., 2007; Yusuf et al., 2010). The increase in the variable fluorescence amplitude was more pronounced in both *A. caudatus* and *A. hypochondriacus* under drought and heat stress. A significant (*p* ≤ 0.05) increase was recorded at the 40°C temperature regime for all species (Figure 3a–h). However, the variable fluorescence amplitude of *A. cruentus* and *A. spinosus* was significantly (*p* ≤ 0.05) lower when compared to *A. caudatus* and *A. hypochondriacus* (Figure 3a–h).

### 3.4 Drought and heat stress effect on photosynthetic efficiency

**FIGURE 4:**
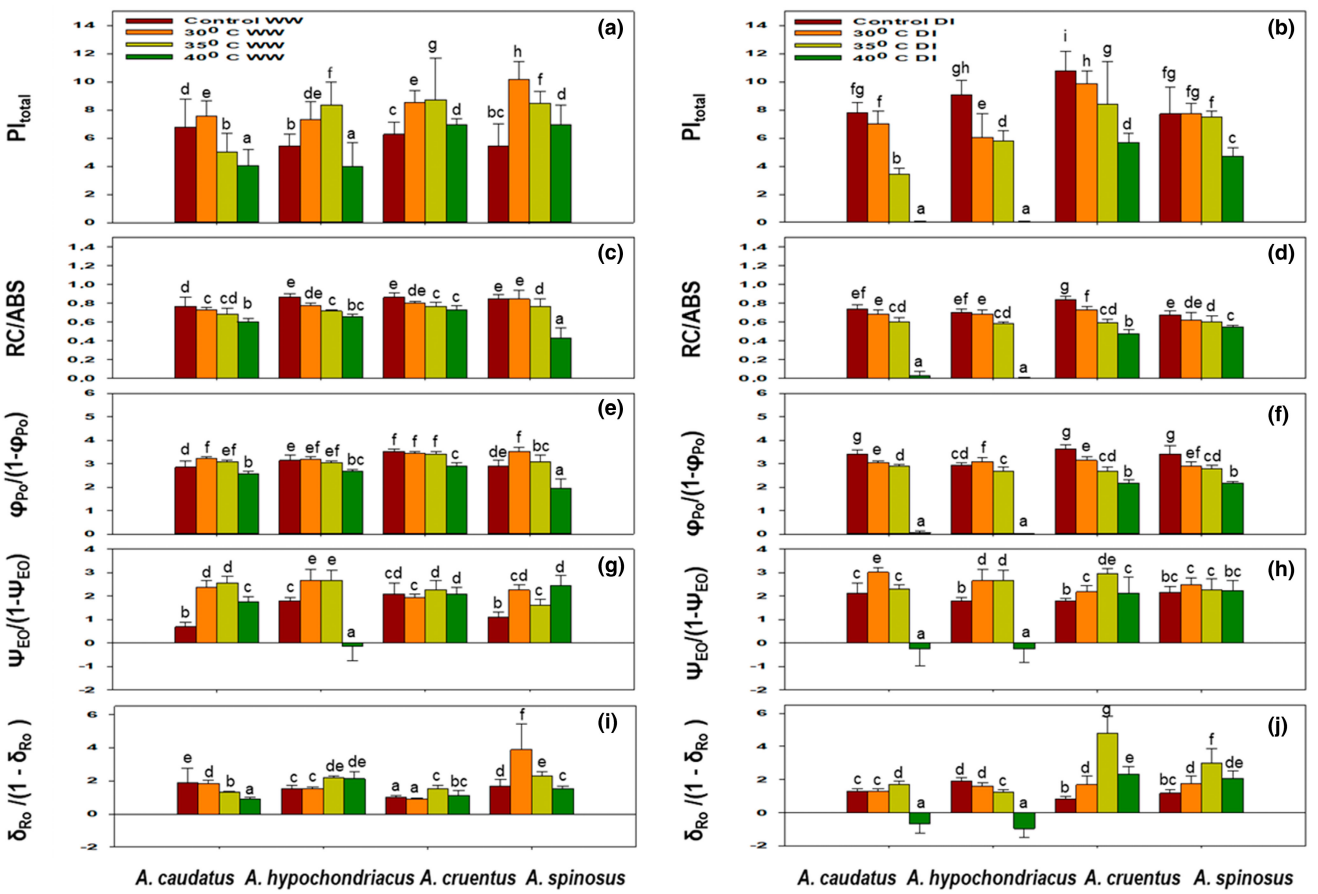
Effects of 30, 35, and 40°C temperature regimes and drought stress on the PI_total_ and RC density on a chlorophyll basis (RC/ABS), the quantum yield of primary photochemistry (*φ*_Po_), the efficiency with which an electron moves into the electron transport chain further than *Q*_A−_ (*ψ*_Eo_) and the probability to reduce the end electron acceptors at the PSI acceptor side (*δ*_Ro_) of *Amaranthus caudatus*, *Amaranthus hypochondriacus*, *Amaranthus cruentus*, and *Amaranthus spinosus* under well‐watered (WW) (a, c, e, g, i), and water‐stressed (DI) condition (b, d, f, h, j). Each vertical bar represents the mean value, and the vertical line is the mean standard error (±) at a 95% confidence level. Values among each species with the same letter(s) are not significantly different at *p* ≤ 0.05.

Total performance index (PI_total_) decreased by ≈50% under well‐watered conditions and ≈100% under drought‐induced conditions for *A. caudatus* and *A. hypochondriacus*, respectively, compared to the control when subjected to a 40°C temperature regime. *Amaranthus cruentus* and *A. spinosus* showed a decrease of 10% and 20%, respectively, under similar conditions (Figure 4a,b). The highest PI_total_ values under drought‐stress conditions were observed in *A. cruentus* and *A. spinosus* (Figure 4b). However, it was significantly (*p* ≤ 0.05) higher in all species at 30 and 35°C temperature regimes under well‐watered conditions and significantly (*p* ≤ 0.05) lower under drought‐induced conditions than in their respective controls (Figure 4a,b).

The parameter RC/ABS representing the reaction center (RC) density on a chlorophyll basis (RC/ABS) was negatively affected by heat stress under water‐stressed conditions. A substantial decrease was observed in *A. caudatus* and *A. hypochondriacus* subjected to a 40°C temperature regime (Figure 4d). However, minor changes were observed under well‐watered conditions for both species and drought‐stress conditions for *A. cruentus* and *A. spinosus* (Figure 4c,d).

The parameter *φ*_Po_/(1 − *φ*_Po_), where *φ*_Po_ is expressing the probability that an absorbed photon will be trapped by the PSII RC and will reduce one *Q*_A_, increased significantly (*p* ≤ 0.05) at 30 and 35°C temperature regimes under well‐watered and drought‐induced conditions in all species (Figure 4e,f). *Acruentus caudatus* and *A. hypochondriacus* showed a substantial decrease in *φ*_Po_/(1 − *φ*_Po_) under drought stress when subjected to a 40°C temperature regime. However, *A. cruentus* and *A. spinosus* showed a significant (*p* ≤ 0.05) increase under similar conditions (Figure 4f).

The efficiency with which an electron moves into the electron transport chain further than *Q*_A−_ (*ψ*_Eo_/[1 − *ψ*_Eo]_), expressed as *ψ*_Eo_ decreased significantly (*p* ≤ 0.05) at 40°C under drought‐induced conditions in *A. caudatus*. A very significant (*p* ≤ 0.05) decrease in *ψ*_Eo_/(1 − *ψ*_Eo_) value was observed for *A. hypochondriacus* at 40°C under all conditions. The decline in *ψ*_Eo_ at 40°C indicates the blockage of electron flow from reduced *Q*_A_ to *Q*_B_ due to heat, drought, and combined stress (Figure 4g,h). In contrast, *A. cruentus* and *A. spinosus* showed a significant (*p* ≤ 0.05) increase in *ψ*_Eo_/(1 − *ψ*_Eo_) under similar conditions. However, all species observed higher values of (*ψ*_Eo_/[1 − *ψ*_Eo_]) at 30 and 35°C, but these values varied significantly under almost all conditions (Figure 4g,h).

The decline in the probability that an electron was transported from reduced PQ to the electron acceptor side of PSI (*δ*_Ro_/[1−*δ*_Ro_]), expressed as *δ*_Ro_ was observed for *A. caudatus* and *A. hypochondriacus* at 40°C under drought stress (Figure 4j). *Acruentus spinosus* showed a substantial increase of *δ*_Ro_/(1−*δ*_Ro_) value at 30°C under well‐watered conditions. In contrast, the highest increase was observed for *A. cruentus* and *A. spinosus* at 35°C under drought‐induced conditions. However, *A. cruentus* showed the lowest increase under well‐watered conditions in all temperature regimes (Figure 4i).

The PSII is sensitive to heat stress, and even short spells of high temperature are known to irreversibly damage the PSII complex (Enami et al., 1994). Drought and heat stress downregulated the photochemical efficiency of PSII. The maximum quantum yield of primary photochemistry (*F*_v_/*F*_m_) decreased significantly (*p* ≤ 0.05) at 40°C in all *Amaranthus* species under well‐watered and drought‐induced conditions (Table 1) when compared to the control. Among the species, the highest decline was observed in *A. caudatus* and *A. hypochondriacus* at 40°C under drought stress (Table 1). The *F*_v_/*F*_m_ was less sensitive to 30 and 35°C temperature regimes under both well‐watered and drought‐induced conditions (Table 1).

**TABLE 1:** The effects of 30, 35, and 40°C temperature regimes under well‐watered (WW) and water‐stressed (DI) conditions on the maximum quantum yield of primary photochemistry (*F*_v_/*F*_m_) of PSII; electron flux reducing end electron acceptors at the PSI acceptor side, per RC (REo/RC); proline; relative water content (%); electrolyte leakage (%) relative to control.

Parameters	Treatments	*Amaranthus caudatus*	*Amaranthus hypochondriacus*	*Amaranthus cruentus*	*Amaranthus spinosus*
*F*_v_/*F*_m_	Control (26°C) WW	0.773 ± 0.010b	0.758 ± 0.013a	0.779 ± 0.005b	0.755 ± 0.018a
	30°C WW	0.762 ± 0.006a	0.762 ± 0.005a	0.775 ± 0.002b	0.778 ± 0.009b
	35°C WW	0.753 ± 0.005a	0.756 ± 0.004ab	0.773 ± 0.005c	0.754 ± 0.018b
	40°C WW	0.720 ± 0.008b	0.727 ± 0.007b	0.743 ± 0.008b	0.654 ± 0.046a
	Control (26°C) DI	0.753 ± 0.017b	0.745 ± 0.007a	0.784 ± 0.007c	0.772 ± 0.021c
	30°C DI	0.752 ± 0.004a	0.756 ± 0.009ab	0.758 ± 0.009b	0.743 ± 0.011a
	35°C DI	0.744 ± 0.004b	0.727 ± 0.015a	0.729 ± 0.013a	0.737 ± 0.010a
	40°C DI	0.055 ± 0.074a	0.008 ± 0.016a	0.684 ± 0.016b	0.683 ± 0.009b
REo/RC	Control (26°C) WW	0.395 ± 0.039c	0.308 ± 0.035b	0.299 ± 0.011ab	0.278 ± 0.019a
	30°C WW	0.362 ± 0.017b	0.306 ± 0.011a	0.474 ± 0.029c	0.385 ± 0.031b
	35°C WW	0.421 ± 0.022ab	0.345 ± 0.051a	0.574 ± 0.020c	0.428 ± 0.077b
	40°C WW	0.491 ± 0.028ab	0.412 ± 0.030a	0.562 ± 0.023b	0.875 ± 0.248c
	Control (26°C) DI	0.351 ± 0.051a	0.534 ± 0.031c	0.371 ± 0.015a	0.417 ± 0.059b
	30°C DI	0.468 ± 0.049a	0.479 ± 0.045ab	0.442 ± 0.078a	0.562 ± 0.083b
	35°C DI	0.532 ± 0.053b	0.421 ± 0.035a	0.636 ± 0.065c	0.551 ± 0.102bc
	40°C DI	0.325 ± 0.208a	0.381 ± 0.143a	0.801 ± 0.145b	0.634 ± 0.025b
Proline	Control (26°C) WW	1037 ± 0.133a	2542 ± 2.389ab	2.561 ± 2.176b	2.680 ± 2.498b
	30°C WW	9280 ± 1.143a	16,078 ± 1.178b	15,687 ± 2.453b	11,956 ± 0.793ab
	35°C WW	9768 ± 3.080a	12,073 ± 0.762b	16,059 ± 2.302c	13,695 ± 2.077ab
	40°C WW	9690 ± 2.260a	7697 ± 0.880a	17,661 ± 0.787b	14,926 ± 0.938b
	Control (26°C) DI	16,879 ± 5.482b	20,044 ± 0.691c	10,198 ± 4.683a	9.944 ± 5.415a
	30°C DI	24,694 ± 1.490c	20,806 ± 1.472b	15,551 ± 2.681a	19,048 ± 7.311bc
	35°C DI	21,607 ± 5.757a	53,822 ± 13.473b	52,493 ± 10,018b	27,194 ± 7.464a
	40°C DI	15,414 ± 2.609a	16,293 ± 3.257a	57,924 ± 7043b	117,496 ± 10,750c
RWC (%)	Control (26°C) WW	86.479	82.990	90.897	88.109
	30°C WW	84.07	80.948	93.079	89.622
	35°C WW	71.454	77.432	91.776	87.368
	40°C WW	71.056	70.775	88.139	86.043
	Control (26°C) DI	43.389	49.797	78.922	72.525
	30°C DI	40.741	43.523	44.396	49.880
	35°C DI	36.709	40.399	40.127	36.204
	40°C DI	33.247	30.701	37.987	35.721
Electrolyte leakage (%)	Control (26°C) WW	30.200	41.500	21.333	21.00
	30°C WW	36.00	49.900	18.667	23.667
	35°C WW	38.100	73.600	25.667	26.00
	40°C WW	50.00	78.600	33.667	26.333
	Control (26°C) DI	54.00	35.800	63.00	30.667
	30°C DI	56.00	42.400	65.00	67.00
	35°C DI	91.00	78.400	70.00	68.00
	40°C DI	93.00	95.00	74.00	71.00

*Note*: Data are shown as mean ± standard deviation and different letters in the same row indicate significant differences (*p* ≤ 0.05).

The specific energy flux extracted from the OJIP test related to the reduction of the final acceptors of the PSI (REo/RC) showed a significant (*p* ≤ 0.05) decrease for *A. caudatus* and *A. hypochondriacus* under drought‐stress conditions in all temperature regimes. In contrast, higher values were observed for *A. cruentus* and *A. spinosus* (Table 1). As the temperature increases, this parameter increased in both species under the well‐watered condition (Table 1). However, no difference in REo/RC was registered between 30 and 35°C temperature regimes in each species under well‐watered and drought‐stress conditions.

Drought and heat stress significantly increased the EL in all *Amaranthus* species (*p* ≤ 0.05). The highest EL was observed in *A. hypochondriacus* (95%) and *A. caudatus* (93%) at 40°C under drought‐stress conditions. In contrast, the lowest EL (26.3%) at 40°C was observed in *A. spinosus* under well‐watered conditions (Table 1). Lower EL values in *A. cruentus* and *A. spinosus* corresponded to the higher values of proline and relative water content (RWC), indicating better protection against oxidative damage induced by drought and heat stress (Giberti et al., 2014; Kaur & Asthir, 2015; Rejeb et al., 2014). The RWC of *A. caudatus* and *A. hypochondriacus* were significantly (*p* ≤ 0.05) higher in all temperature regimes under well‐watered conditions (Table 1). In contrast, a decrease of ≈53% was observed under drought‐stress conditions (Table 1). It was also observed that these species had lower proline values compared to *A. cruentus* and *A. spinosus* under similar conditions (Table 1).

## 4 DISCUSSION

*Amaranthus* has the ability to repair damaged tissues after heat stress exposure. It can resume normal metabolic functions, such as photosynthesis, faster than other non‐heat tolerant leaf vegetables which have earned it the status of being heat‐tolerant (Gou & Al‐Khatib, 2003; Moran & Showeler, 2005; Paland & Chang, 2003). It is also well known that heat stress has adverse effects on PSII, PSI, and other electron transport chain components (ETC) (Mathur et al., 2011; Tiwari et al., 2008; Živčák et al., 2015). The fluorescence parameters indicated that the photosynthetic apparatus of *A. cruentus* and *A. spinosus* were more tolerant to drought and heat stress compared to *A. caudatus* and *A. hypochondriacus* (Figure 4a–j and Table 1). However, its response varied according to the type and severity of the stress in both species.

Changes in the OJIP curve depended on the temperature regime, decreasing in parallel with increasing temperature (Figure 1a–h). These changes in the fluorescence intensity were associated with the restriction in the flow of electrons between PSII and PSI, beyond *Q*_A_, and decreased plants' ability to reduce NADP^+^ to NADPH (Oukarroum et al., 2012). The shape of the OJIP‐transient was influenced by drought and heat stress (Figure 1a–h), indicative of controlled PSII to PSI electron flow (Aksmann & Tkaj, 2008). The insignificant changes observed at the O step (Figure 1a–h) suggest slight or no loss of energy transfer to the RCs from the antenna complex. Simultaneously, a higher number of inactive RCs can also result in an O step with a lower peak (Figure 1e–h). This can prevent the electrons from being transferred from reduced *Q*_A_^−^ or light‐harvesting complex II (LHCII) to PSII core (Baker, 2008; Murkowski, 2002). The variation in PSII temperature tolerance between *Amaranthus* species observed in this experiment was consistent with the results in barley landraces and varieties (Kalaji et al., 2018; Oukarroum et al., 2009, 2016), and field‐grown winter wheat genotypes (Brestic et al., 2012) exposed to drought and heat stress.

A positive ∆*V*_L_‐band observed in this study from plants subjected to 30, 35 and 40°C temperature regimes under both conditions (Figure 2a–h) suggested that drought and heat stress made the energetic cooperation among the PSII units to be less stable (Pollastrini et al., 2017). This further indicated that drought and heat stress caused a change in the thylakoid membrane structure (Oukarroum et al., 2007). Similarly, a positive ∆*V*_K_‐band, such as those observed in both species treated with 30, 35, and 40°C under both well‐watered and drought‐induced conditions (Figure 3a–h), indicated that the efficiency of the OEC to split water and provide electrons to a *P*_680_ RC was reduced (Oukarroum et al., 2012). A positive ∆*V*K‐band may indicate an increased antennae size of the PSII (Yusuf et al., 2010) and disruption between the donor side and the acceptor side of the PSII. This could cause an imbalance of electron flow between the OEC to the RC and the acceptor side of the PSII to PSI (Chen & Cheng, 2010; Strasser, 1997). This suggests that drought and heat stress have weakened the OEC's stability (Gururani et al., 2012, 2015; Kalaji et al., 2014). The alteration in the OEC enabled alternative electron donors such as proline that circumvent OEC dissociation by transferring electrons to the PSII, which led to an increase in the Pheo^−^ and *Q*_A_^−^, and the generation of a positive ∆*V*_K_‐band (De Ronde et al., 2004; Strasser et al., 2000).

The values of the maximum quantum yield of the primary photochemistry (*F*_v_/*F*_m_) of PSII are regarded as a sensitive indicator of the plant's photosynthetic performance exposed to heat stress (Li et al., 2010). A decline in the *F*_v_/*F*_m_ values can be used to identify damaged PSII structure (Badr & Brüggermann, 2020). However, the *F*_v_/*F*_m_ value of 0.750 is considered a boundary value for a fully functional PSII (Strasser et al., 2004). Drought and moderate heat (30 and 35°C) stress had an insignificant effect on the potential quantum efficiency (*F*_v_/*F*_m_) of PSII (Table 1). This confirmed the high stability of the potential PSII photochemical efficiency (Oukarroum et al., 2007) during drought and moderate heat stress. It was also noted that when the temperature was at 40°C, there was a substantial decrease below the boundary value of this parameter under drought stress in *A. caudatus* (69.8%) and *A. hypochondriacus* (73.7%; Table 1). This was an indication of the inhibition of the PSII activity, increased energy dissipation, disruption of the PSII antenna complex, and impaired regeneration ability of ribulose‐1,5‐biphosphate (RuBP) (Rai & Agrawal, 2008; Vieira Santos et al., 2001). Thus, the damage to the PSII structure by drought and severe heat stress blocked the photosynthetic ETC connecting the PSII and PSI, which has led to the reduction of the *F*_v_/*F*_m_ ratio (Goltsev et al., 2016; Rai & Agrawal, 2008; Sun et al., 2016; Zaghdoudi et al., 2011).

The decrease in REo/RC for *A. caudatus* and *A. hypochondriacus* under drought‐stress conditions for all temperature regimes (Table 1) could be attributed to a decreased electron capacity between *Q*_A_^−^ and PSI acceptors. However, the increase observed in *A. cruentus* and *A. spinosus* (Table 1) under similar conditions could infer an increase in the I‐P phase to the OJIP transient (Figure 1g,h) which is genotype‐dependent. This further indicates that there was an increased pool size of the final electron acceptors of PSI (Tsimilli‐Michael & Strasser, 2008; Živčák et al., 2014) in *A. cruentus* and *A. spinosus*. Similar results were reported by Oukarroum et al., 2009 and Umar et al., 2019 on barley varieties and sunflower cultivars, respectively. The increase in this parameter as the temperature increased in *A. cruentus* and *A. spinosus* indicated that heat stress under both conditions (Table 1) did not disrupt the electron transport beyond *Q*_A_^−^ to the intersystem of electron acceptors. These further suggest that drought and heat stress activated adaptive mechanism in *A. cruentus* and *A. spinosus*.

In this study, drought and heat (at 40°C) stress caused a decrease in the photochemical quantum yield of the PSII in *A. caudatus* and *A. hypochondriacus* (Figure 4f). Consequently, a decrease in *φ*_Po_ indicated a damaged PSII complex due to photoinhibition (Baker & Rosenqvist, 2004). Furthermore, this disruption reduces the efficiency with which an absorbed photon could be captured by the RC of the PSII (Oukarroum et al., 2009). Besides, the decline in *F*_v_/*F*_m,_ RC/ABS, *φ*_Po,_ *ψ*_Eo_, and *δ*_Ro_ values further supported the occurrence of photoinhibition in *A. caudatus* and *A. hypochondriacus* (Figure 4c–j and Table 1), as it indicated a blockage in the electron transfer across the intersystem to the final acceptors of PSI (Zhuo et al., 2017). On the contrary, higher values of *F*_v_/*F*_m_, RC/ABS, *φ*_Po_, *ψ*_Eo_, and *δ*_Ro_ were observed for *A. cruentus* and *A. spinosus* under similar conditions (Figure 4c–j and Table 1), suggesting the stability of the reduction of PSI final acceptors, PSII structure, and intersystem (Oukarroum et al., 2009; Strasser et al., 2004; Tsimilli‐Michael & Strasser, 2008; Xue et al., 2017). Therefore, *A. caudatus* and *A. hypochondriacus* lack adaptive mechanisms to tolerate drought and heat stress. In contrast, *A. cruentus* and *A. spinosus* adapted by converting active RCs into inactive RCs which reduced light absorption on the chlorophyll basis (RC/ABS), trapping efficiency (*φ*_Po_), and PSII activity. The decrease resulted in the downregulation of PSII preventing the electrons from being transferred between the RCs and reduced *Q*_A_^−^ (*ψ*_Eo_; Figure 4c–j).

The PI_total_ is considered the most sensitive parameter of the OJIP‐test and an efficient tool to quantify plant stress. It takes into account the performance of partial energy conservation from photons absorbed by PSII to the reduction flux (RE) of PSI end electron acceptors (Strasser et al., 2010; Tsimilli‐Michael & Strasser, 2008; Yusuf et al., 2010). Other C4 species can tolerate temperatures up to 45°C (Mingnan et al., 2017). In this study, there was irreversible damage to the PSII structure observed at 40°C under drought stress on *Amaranthus* species tested (Figure 4d,f,g,h,j and Table 1). Both *A. caudatus* and *A. hypochondriacus* showed higher sensitivity to drought and heat stress, as the PI_total_ decreased by 50% and 100%, respectively, at 40°C. In contrast, *A. cruentus* and *A. spinosus* decreased by 10% and 20%, respectively, under similar conditions (Figure 4a,b). Higher values of PI_total_ observed in *A. cruentus* and *A. spinosus* indicated the photosynthetic apparatus' ability to increase the potential energy conservation (Yusuf et al., 2010). This is a confirmation of the better performance of the PSI acceptor side in *A. cruentus* and *A. spinosus* as compared to *A. caudatus* and *A. hypochondriacus*. The PI_total_ is a multiparametric expression that links *F*_v_/*F*_m,_ RC/ABS, *φ*_Po_, and *ψ*_Eo_. It also encompasses the increase in the prospect that an electron from the intersystem (*δ*_Ro_) moves to reduce the final acceptors on the acceptor side of the PSI (Chen et al., 2015; Krüger et al., 2014; Redillas et al., 2011; Strasser et al., 2010; Tsimilli‐Michael & Strasser, 2008; Yusuf et al., 2010). It was also noted that *F*_v_/*F*_m,_ RC/ABS, *φ*_Po_, *ψ*_Eo_, and *δ*_Ro_ were all sensitive to heat (at 40°C) and the combined effect of drought and heat stress (Figure 4d,f,h,j and Table 1), though *F*_v_/*F*_m,_ *φ*_Po_ and *δ*_Ro_ were less sensitive to heat stress under well‐watered conditions (Figure 4e,i and Table 1). Further, the most sensitive parameter of the electron transport chain to a combination of drought and heat stress appeared to have been the probability that an absorbed photon will be trapped by the PSII RC and will reduce one *Q*_A_ (*ψ*_Eo_) and the RC density on a chlorophyll basis, which decreased by 70.3% and 69.7% in *A. caudatus* and *A. hypochondriacus*, respectively (Figure 4d,h). These observations directly suggested that the higher decline under drought and heat stress indicated that these stressors damaged the photosynthetic apparatus of these species. In contrast, a significant increase in *δ*_Ro_ observed in *A. cruentus* and *A. spinosus* under combined stress was an indication of efficient electron transfer toward the end electron acceptors of PSI.

Simultaneous environmental stresses like drought and heat disturb plant‐water relations and disrupt and damage cell membranes, altering their permeability. In response to the stress, plants divert protein synthetic machinery toward proline accumulation, which improves plant water relations and membrane structure and stability (Claussen, 2005; Kaur & Asthir, 2015; Rejeb et al., 2014; Slathia et al., 2012). However, proline can be toxic to cells when plants are exposed to a combination of drought and heat stress. This happens as a result of alteration in the balance of proline biosynthesis and degradation by heat stress, causing the accumulation of Δ^1^‐pyrroline‐5‐carboxylate (P5C) and other intermediates in the mitochondria (Giberti et al., 2014; Mani et al., 2002; Rizhsky et al., 2002, 2004). Out of four species treated with 30, 35, and 40°C temperature regimes, *A. cruentus* and *A. spinosus* had a higher proline and lower RWC and EL content than *A. caudatus* and *A. hypochondriacus* under well‐watered and drought‐stress conditions (Table 1).

Accumulation of proline by *A. cruentus* and *A. spinosus* is an acclimation strategy resulting from the inactivation of the OEC and constrained electron flow to the PSII. To enhance tolerance to drought and heat stress proline act as an alternative electron donor that circumvent OEC dissociation by transferring electrons to the PSII, which led to an increase in the Pheo^−^ and *Q*_A_^−^, and generation of a positive ∆*V*_K_‐band (De Ronde et al., 2004; Strasser et al., 2007; Zhou et al., 2019). This vital acclimation response protects the photosynthetic machinery against oxidative damage, ensuring the plant's ability to reduce NADP^+^ to NADPH (*δ*_Ro_). A significant decrease of proline in *A. caudatus* and *A. hypochondriacus* under combined stress resulted in the downregultion of PSII, which has led to the reduction of *F*_v_/*F*_m,_ RC/ABS, *φ*_Po_, *ψ*_Eo_. This has influenced the reduction of electron transfer across the intersystem (*δ*_Ro_) to the PSI final acceptors and damage to the PSII structure (Figure 4c–j and Table 1). Decreased RWC can cause loss of cellular functioning (Conde et al., 2011). Higher EL in *A. caudatus* and *A. hypochondriacus* (Table 1) was an indication of their membrane instability, which could be due to degradation in the lipid‐protein configuration and loss of cellular functioning (Conde et al., 2011; Earnshaw & Hendrey, 1993) under heat and combined stress. The reduction in RWC and EL due to combined stress contributed to the decrease in maximum quantum yield of the primary photochemistry (*F*_v_/*F*_m_) and inhibition of PSII activity (Lu & Zhang, 1999; Killi et al., 2017) in *A. caudatus* and *A. hypochondriacus*.

## 5 CONCLUSIONS

It was evident from this investigation that the ability to tolerate heat and drought stress varies between the species studied. Our results indicated that *A. cruentus* and *A. spinosus* have better adaptive mechanisms to tolerate drought and heat stress than *A. caudatus* and *A. hypochondriacus*.

This study demonstrated that photosynthetic apparatus, RWC and EL of *Amaranthus* species were not affected by moderate heat stress (30 and 35°C). All species had a high‐temperature tolerance level. The ∆*V*_L_ and ∆*V*_K_‐bands are good indicators to identify the physiological disruptions in the PSII before the appearance of the visual damage caused by the stress.

Among the discussed parameters, PI_total_ was more sensitive to different temperature regimes and drought stress, as it represents the overall behavior of PSII. Proline is a reliable indicator when assessing the effects of drought and heat stress on *Amaranthus*. Besides, detailed drought and heat stress tolerance studies of these species' cultivars remain necessary to correctly identify cultivars suitable for arid and semi‐arid regions in the SADC region.

## CONFLICT OF INTEREST

The authors declare that there is no conflict of interest.

## Supporting information


Appendix S1

## Data Availability

The data that support the findings of this study are openly available in [Boloka: NWU Institutional Repository] at [https://repository.nwu.ac.za/].
