# Supplementary material for: The interactive effects of drought and heat stress on photosynthetic efficiency and biochemical defense mechanisms of Amaranthus species
Source: Plant Environ Interact. 2022 Oct 13;3(5):212–25. doi: 10.1002/pei3.10092 (PMC10168097; doi:10.1002/pei3.10092)
Supplement: Supplementary file 1 — Appendix S1 [file PEI3-3-212-s001.docx]

**Appendix A. Summary of the JIP-test formulae and terms (Courtesy of Strasser *et al*., 2004)**

| Extracted data from the recorded fluorescence transient OJIP | | |
| --- | --- | --- |
| F_t_ | | fluorescence at time t after onset of actinic illumination |
| F_20μs_ | | minimal reliable recorded fluorescence 20 μs with the Handy-PEA-fluorimeter |
| F_100μs_ | | fluorescence intensity at 100μs |
| F_300μs_ | | fluorescence intensity at 300μs |
| F_J_ | | fluorescence intensity at the J-step (2 ms) of OJIP |
| F_I_ | | fluorescence intensity at the I-step (30 ms) of OJIP |
| F_P_ | | maximal recorded fluorescence intensity, at the peak P (300ms) of OJIP |
| t_FM_ | | time (ms) to reach the maximal fluorescence intensity FM |
| V_J_ | | relative variable fluorescence at the J-step = (F_2ms_-F_o_)/ (F_M_-F_o_) |
| Area | | total complementary area between the fluorescence induction curve and F = F_M_ |
| Quantum yields or flux ratios or efficiencies | | |
| *ϕ*P_o_=TR_o_/ABS=[1−(*F*_o_/*F*_M_)]=*F*_V_/*F*_M_ | | maximum quantum yield for primary photochemistry |
| *ϕ*E_o_=ET_o_/ABS=[1−(*F*_o_/*F*_M_)]·*Ψ_E_*_o_ | | quantum yield for electron transport (ET) |
| *Ψ*_o_ =ET_o_/TR_o_ = (1−*V*_J_) | | efficiency/probability for electron transport (ET) |
|  | | **Specific energy fluxes or activities** |
| ABS/RC =*M*_o_·(1/*V*_J_)·(1/*ϕ*_Po_) | | absorption flux (of antenna Chlorophylls) per RC |
| TR_o_/RC =*M*_o_·(1/*V*_J_) | | trapped energy flux (leading to QA reduction) per RC |
| ET_o_/RC =*M*_o_·(1/*V*_J_)·*Ψ*_o_ | | electron transport flux (further than QA |
| DI_o_/RC = (ABS/RC)−(TR_o_/RC) | | dissipation energy flux at the level of the antenna chlorophylls |
| RE_0_/RC = M_0_ (1/V_J_)ψ_Eo_ δ_Ro_ | | electron flux reducing end electron acceptors at the PSI acceptor side, per RC |
| Phenomenogical fluxes or activities | | |
| ABS/CS = ABS/CSChl = Chl/CS or ABS/CS_o_ = *F*_o_ or ABS/CS_M_ =*F*_M_ | | absorption per excited cross-section |
| TR_o_/CS = *ϕ*_Po_·(ABS/CS) | | trapping per excited cross-section |
| ET_o_/CS = *ϕ*_Po_·*Ψ*o·(ABS/CS) | | electron transport per excited cross-section |
| DI_o_/CS = (ABS/CS)−(TR_o_/CS) | | dissipation per excited cross-section |
|  | | **Density of reaction centers** |
| RC/CS = ϕPo•(VJ/Mo)•ABS/CS | fraction of active reaction centres per excited cross-section of leaf | |
|  | | **Performance indices** |
| PI_ABS_ = [γ_RC/_(1-γ_RC_)]. [φ_Po_/(1- φ_Po_)].[ψ_o_/(1- ψ_o_] | performance index (potential) for energy conservation from exciton to the reduction of intersystem electron acceptors | |
| PI_total_ =(PI_ABS_).(δ_Ro_/1- δ_Ro_) | | performance index (potential) for energy conservation from exciton to the reduction of PSI end acceptors |
